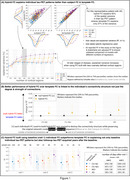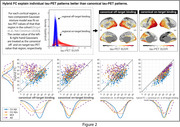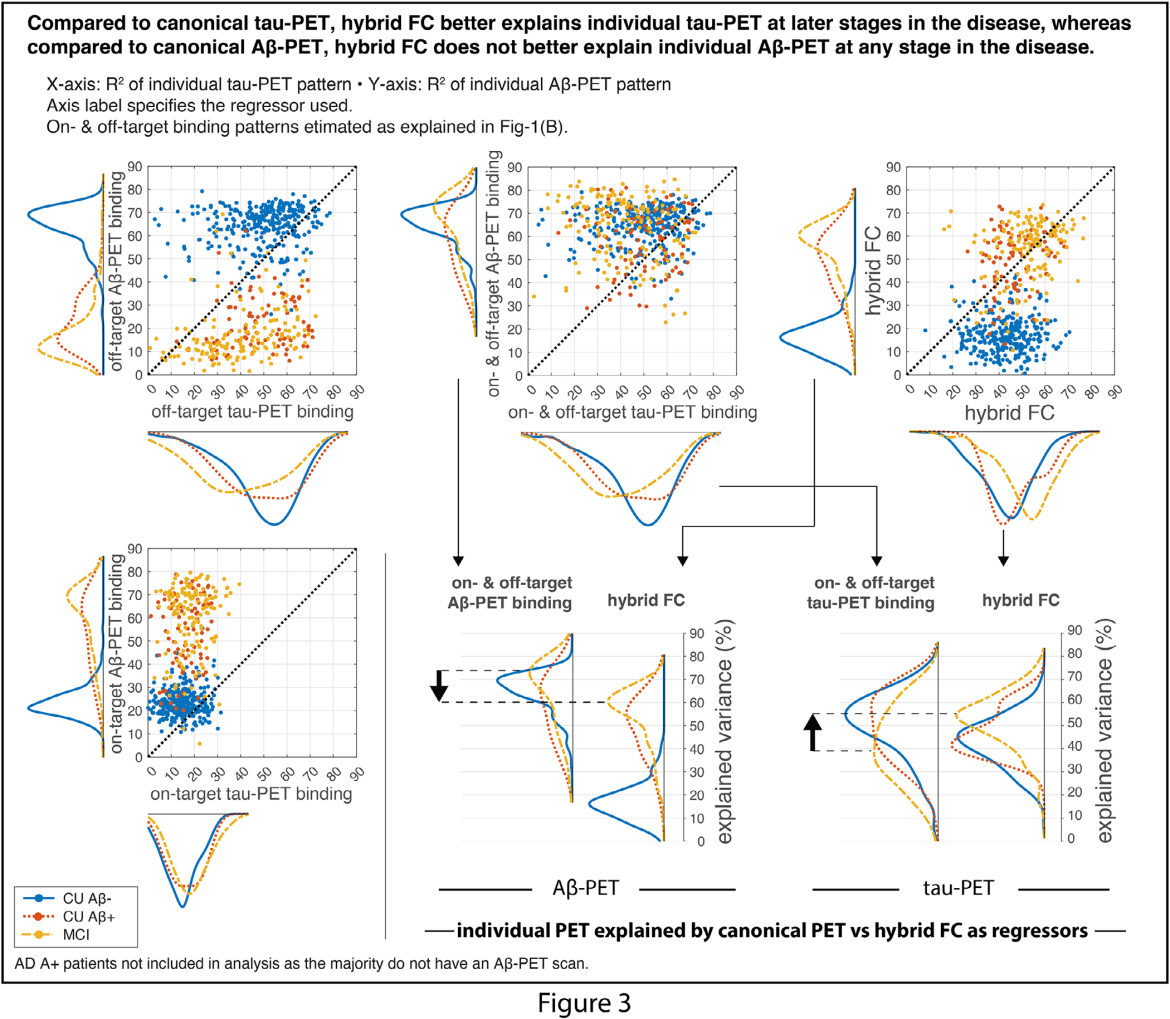# Individual functional connectivity constraints on spatial progression of tau pathology in Alzheimer's disease

**DOI:** 10.1002/alz70856_104872

**Published:** 2026-01-08

**Authors:** Harry H Behjat, Jacob W. Vogel, Olof Strandberg, Nicola Spotorno, Jonathan Rittmo, Lyduine E. Collij, Alexa Pichet Binette, Yu Xiao, Danielle van Westen, Erik Stomrud, Sebastian Palmqvist, Niklas Mattsson‐Carlgren, Dimitri Van De Ville, Ruben Smith, Oskar Hansson, Rik Ossenkoppele

**Affiliations:** ^1^ Clinical Memory Research Unit, Lund University, Lund, Sweden; ^2^ Department of Clinical Sciences Malmö, Faculty of Medicine, SciLifeLab, Lund University, Lund, Sweden; ^3^ Clinical Memory Research Unit, Department of Clinical Sciences Malmö, Faculty of Medicine, Lund University, Lund, Sweden; ^4^ Radiology and Nuclear Medicine, Vrije Universiteit Amsterdam, Amsterdam UMC, Amsterdam, Netherlands; ^5^ Amsterdam Neuroscience, Brain Imaging, Amsterdam, Netherlands; ^6^ Department of Physiology and Pharmacology, Université de Montréal, Montréal, QC, Canada; ^7^ Centre de Recherche de l’Institut Universitaire de Gériatrie de Montréal, Montréal, QC, Canada; ^8^ Diagnostic Radiology, Department of Clinical Sciences Lund, Lund University, Lund, Sweden; ^9^ Memory Clinic, Skåne University Hospital, Malmö, Skåne, Sweden; ^10^ Wallenberg Center for Molecular Medicine, Lund University, Lund, Sweden; ^11^ Neuro‐X Institute, Ecole Polytechnique Fédérale de Lausanne (EPFL), Geneva, Switzerland; ^12^ Department of Radiology and Medical Informatics, University of Geneva, Geneva, Switzerland; ^13^ Clinical Memory Research Unit, Lund University, Malmö, Skåne, Sweden; ^14^ VU University Medical Center, Amsterdam UMC, Amsterdam, Netherlands

## Abstract

**Background:**

Tau PET patterns show notable spatial heterogeneity across subjects in Alzheimer's disease (AD). In vitro findings suggest that tau may spread ‘prion‐like’ across neuronal connections in an activity‐dependent manner, a hypothesis strengthened by group‐level studies showing association between resting‐state functional connectivity (FC) networks and tau deposition patterns. This hypothesis would be better supported by evidence at the individual level, an investigation that is the focus of this study.

**Method:**

Structural MRI, resting‐state fMRI, tau‐PET, and amyloid‐β (Aβ)‐PET data from 733 participants aged 50+ from the BioFINDER‐2 study were used: 402 cognitively unimpaired (CU, CSF Aβ+=89), 157 with mild cognitive impairment (MCI), and 174 with AD dementia, with 523 follow‐up tau‐PET scans (323 CU, 109 MCI, 91 AD); individuals with MCI or AD dementia were all CSF Aβ+. fMRI data were pre‐processed, and surface parcellated in subject‐space. To reduce instability in subject‐level estimations while retaining individual information, template FC (CU Aβ‐ group‐average) and each participant's individual FC were used to build an individualised ‘hybrid’ FC; template and subject‐specific regional FC profiles were integrated by statistically estimating the contribution of each in explaining the participant's tau‐PET. FC‐based results were compared against using canonical PET patterns estimated from the distribution of regional PET values across the cohort.

**Result:**

Hybrid (i.e. individualized) FC explained tau‐PET patterns better than template FC across the continuum (Fig‐1A), which exceeded chance based on null modeling (Fig‐1B). Baseline hybrid FC also explained follow‐up tau‐PET patterns better than template FC (Fig‐1C). For individuals with MCI or AD dementia, hybrid FC alone explained tau better than enforcing canonical tau patterns on everyone (Fig‐2), whereas canonical patterns better explained the data of individuals at early stages of the disease. However, in contrast to tau‐PET patterns, individual Aβ‐PET patterns–for which prion‐like spread hypothesis was not assumed—were not better explained by hybrid FC than by Aβ‐PET canonical patterns (Fig‐3).

**Conclusion:**

Our results provide compelling implicit evidence in support for the hypothesis of tau spread via communicating neurons at the individual‐level. These findings strengthen the potential of using brain network‐based models for sample stratification in AD clinical trials and prognosis in clinical practice.